# Beyond the Binding Site: The Role of the β2 – β3 Loop and Extra-Domain Structures in PDZ Domains

**DOI:** 10.1371/journal.pcbi.1002429

**Published:** 2012-03-08

**Authors:** Stefano Mostarda, David Gfeller, Francesco Rao

**Affiliations:** 1 Freiburg Institute for Advanced Studies, School of Soft Matter Research, Freiburg im Breisgau, Germany; 2 Molecular Modeling, Swiss Institute of Bioinformatics, Lausanne, Switzerland; National Cancer Institute, United States of America and Tel Aviv University, Israel, United States of America

## Abstract

A general paradigm to understand protein function is to look at properties of isolated well conserved domains, such as SH3 or PDZ domains. While common features of domain families are well understood, the role of subtle differences among members of these families is less clear. Here, molecular dynamics simulations indicate that the binding mechanism in PSD95-PDZ3 is critically regulated via interactions outside the canonical binding site, involving both the poorly conserved 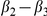 loop and an extra-domain helix. Using the CRIPT peptide as a prototypical ligand, our simulations suggest that a network of salt-bridges between the ligand and this loop is necessary for binding. These contacts interconvert between each other on a time scale of a few tens of nanoseconds, making them elusive to X-ray crystallography. The loop is stabilized by an extra-domain helix. The latter influences the global dynamics of the domain, considerably increasing binding affinity. We found that two key contacts between the helix and the domain, one involving the 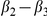 loop, provide an atomistic interpretation of the increased affinity. Our analysis indicates that both extra-domain segments and loosely conserved regions play critical roles in PDZ binding affinity and specificity.

## Author Summary

Protein interactions play crucial roles in all biological processes. A common way of studying them is to focus on sub-parts of proteins, called domains, that mediate specific types of interactions. For instance, it is known that most PDZ domains mediate protein interactions by binding to the C-terminus of other proteins. Humans have more than 200 slightly different copies of these domains. At the level of the binding site, PDZ domains look quite similar. This is in apparent contradiction with their heterogeneous binding specificity. Using detailed molecular dynamics simulations in conjunction with statistical analysis, we predict that contacts outside of the canonical binding site play important roles in regulating protein interactions. Some of these contacts influence the overall dynamics of PDZ domains, providing an explanation for their allosteric effect. These interactions involve regions of the PDZ domains that are much less conserved, suggesting that they can help in differentiating selectivity in this large domain family.

## Introduction

PDZ domains are modular protein interaction domains specialized in binding short linear motifs at the C-terminus of their cognate protein partners [1], [2]. In human, they are found in hundreds of different proteins and are mostly involved in cell-cell adhesion and epithelial junctions [3]. PDZ domains are often classified on the basis of their preferred C-terminal ligand sequences. Early studies organized binding specificity in three canonical classes: class-I involving C-terminal motifs of the type [x–(s/t)–x–(v/i)_cooh_], class-II [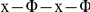
_cooh_] and class-III [x–(d/e)–x–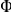
_cooh_], where 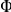 is a hydrophobic residue and x any amino acid [2], [4]. This classification, though consistent with the highly conserved binding groove [2], does not explain the large selectivity observed both in naturally occurring C-terminal peptides and synthetic peptide library screening [5]–[8]. Systematic investigations of PDZ domain specificity revealed that more distal C-terminal peptide residues are involved in the binding process [7], [9], suggesting a role for the 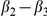 loop following the binding site [10]–[15]. For example, the solution structure of the second domain of the hPTP1E protein showed that this loop interacts with the sixth amino acid from the peptide C-terminus [10], while possible electrostatic contacts between the loop and peptide amino acids up to position eight were found in the Par3 PDZ3-VE-Cad domain [14], [15].

It was recently suggested that specificity beyond the canonical classes can be obtained by long-range interactions involving non-conserved structural motifs specific to the domain [16]. For instance, the extra-domain helical extension characterizing the third PDZ domain of PSD95 (also called DLG4 or SAP90) was shown to influence binding [17]. Although this helix is away from the binding groove, affinity is reduced by 21-fold upon truncation of this non-conserved structural motif. Titration calorimetry measurements indicated that the free-energy penalty is entropic in nature. It was proposed that enhanced side-chain flexibility upon helix truncation, which is subsequently quenched by peptide binding, might be the main reason for this effect. This exquisitely dynamical behavior, calling for a hidden dynamic allostery [17], [18], pinpointed the importance of conformational entropy upon binding mediated by structural elements not directly evident from structural inspection alone [19], [20].

Here, we investigate the set of interactions beyond the binding site influencing peptide binding in the PSD95-PDZ3:CRIPT complex. Molecular dynamics (MD) simulations indicate that residues upstream of the 4th C-terminal amino acid are crucial for binding. Specifically, lysines residues at position −4 and −7 in the CRIPT peptide are observed to dynamically interact with the 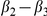 loop. Shorter peptides spontaneously unbind from the domain, indicating that canonical interactions within the binding site are not sufficient for binding. Further simulations of the DLG1-PDZ2:E6 complex suggest a wide spread presence of such peptide-loop interactions in the PDZ family. Finally, we find that the extra-domain helix of PSD95-PDZ3 helps stabilizing the 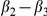 loop via ionic interactions. Our results provide direct evidence of the role played by peptide amino acids away from the C-terminus and the interplay with previously unrecognized PDZ structural motifs.

## Results

### Protein-Ligand contacts beyond the binding site

**Figure 1:**
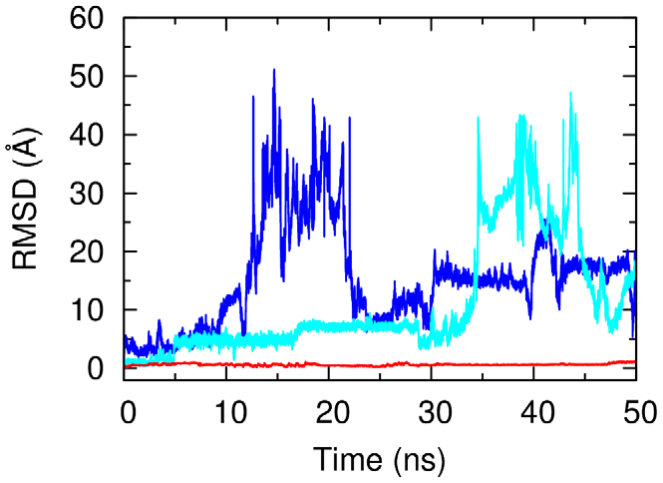
Time series of backbone RMSD from the crystal structure for the CRIPT peptide along MD simulations (residues 0:−4, first 50 ns). Blue and light-blue curves show two sample unbinding trajectories of the 5-mer peptide CRIPT5. The red curve shows the time series for the longer 9 residues peptide CRIPT9.

Seminal X-ray crystallography experiments on the third PDZ domain of PSD95 in complex with the CRIPT C-terminal peptide indicated that peptide binding is realized through the last four residues (peptide positions 0 to −3), while the rest of the peptide is mostly disordered [1] (the system was crystallized with a 9-mer peptide, see below). This observation suggested a minor role of residues upstream of the last four ones for binding. To test this hypothesis, four MD simulation runs were carried out using a 5-mer peptide from CRIPT (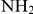-KQTSV-COOH, CRIPT5), a natural class-I binder of PSD95-PDZ3 (see Methods) [1], [17]. Unexpectedly, all the four runs showed spontaneous unbinding within the first 110 ns (see blue and light-blue lines of Fig. 1 for two unbinding trajectories and Table S1 for specific unbinding times and simulation lengths). Weak affinity was a somewhat surprising result, suggesting that canonical class-I interactions alone are not sufficient for binding. Interestingly, one of the runs showed rebinding from a partially unbound state. This event was mediated by the interaction of 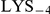 on the peptide with 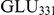 on the 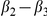 loop following the binding site as shown in Fig. S1. The same peptide with a charged N-terminus (CRIPT5*), which can reinforce this type of electrostatic interactions, remained anchored to the binding site for the total simulation time [21]. However, the peptide canonical contacts were only partially formed (see Fig. S2).

These observations suggested that interactions beyond the canonical class-I motif are needed to achieve stable binding in native conditions (i.e. without an artificially charged N-terminal peptide), possibly with a major role of the 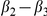 loop. To elucidate this point, four simulations with a longer 9-mer CRIPT peptide (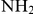-TKNYKQTSV-COOH, CRIPT9) were performed for a total of roughly 700 ns. The peptide remained bound to the original X-ray configuration in all runs (see red curve in Fig. 1 for a typical RMSD time trace). Strikingly, the four extra amino acids strongly influenced binding. The two lysines at peptide positions −4 and −7 transiently formed specific salt-bridges with two negatively charged loop residues, 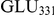 and 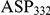. These contacts are dynamic, interconverting between each other on the ns time scale. On the other hand, their cumulative contribution is large: the loop and the ligand are in contact via salt-bridges for 44% of the time. These results indicate an unexpected and biologically relevant role of this loop, going beyond class-I interactions.

**Figure 2:**
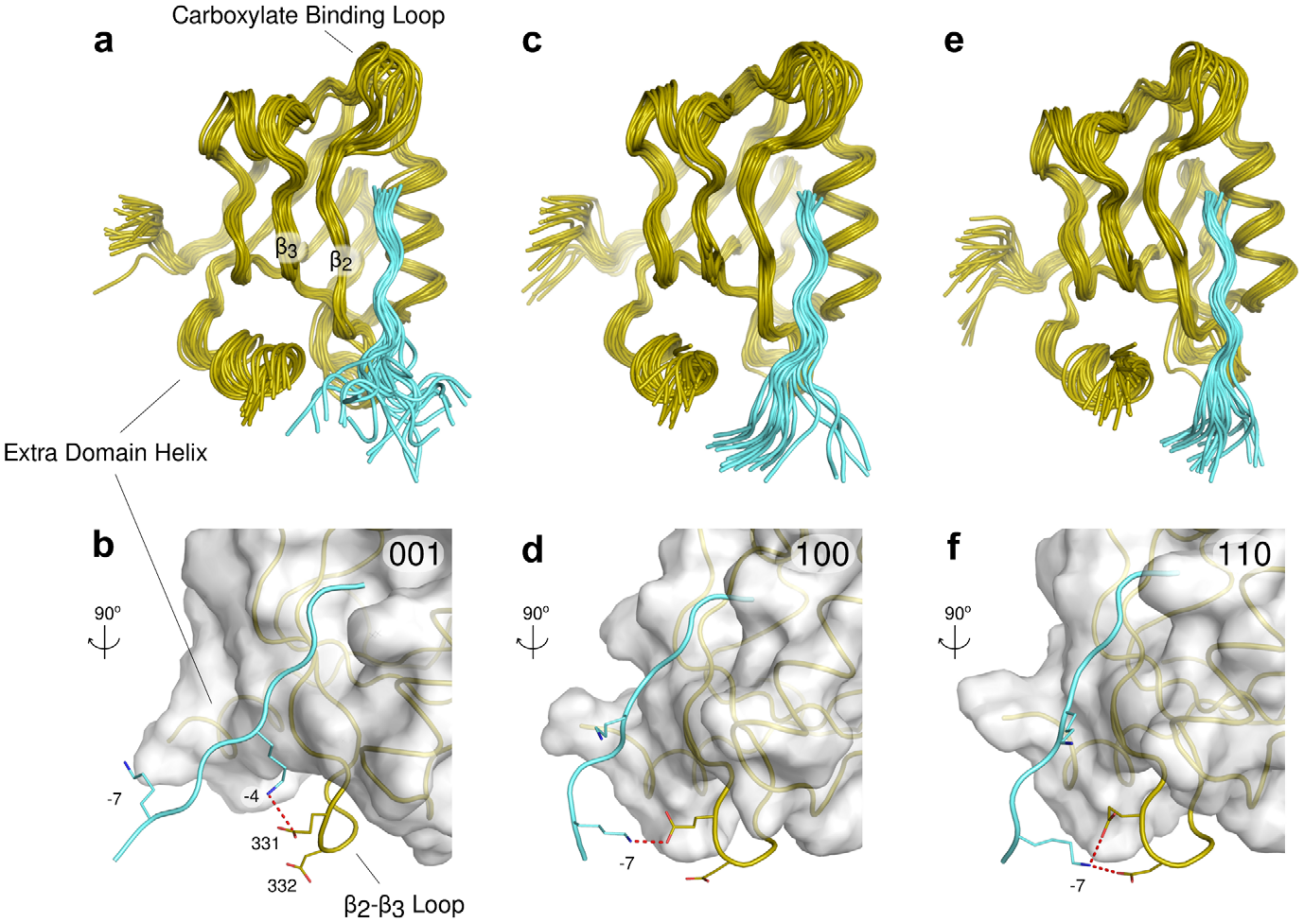
The three most populated binding modes of the 9-mer CRIPT peptide to the wild-type PSD95-PDZ3 domain. Specific ionic interactions between the peptide and the 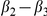 loop are displayed in panels (b), (d) and (f). (a–b) 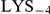 interacting with 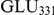 of the 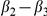 loop. (c–d) 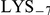 interacting with 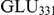. (e–f) 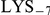 interacting with both 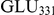 and 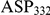. The strings “001”, “100” and “110” encode the interaction patterns (see Results).

Structural cluster analysis provides a quantitative classification of the non-canonical interactions (see Methods for details). In Fig. 2, structural ensembles characterizing the three most populated peptide-loop configurations are shown. We used a simplified code to classify the peptide-loop interactions. At the first, second and third position there is a “1” if interactions −7∶331, −7∶332 or −4∶331 are formed, respectively; “0” otherwise (these three contacts are the statistically more relevant ones). For example, “110” indicates that peptide 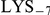 is in contact with both 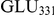 and 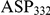, as shown in Fig. 2e–f. The most observed configurations are “110”, “001” and “100”, having a relative population of 13%, 10% and 8%, respectively (see Fig. 2 for their structural characterization; the cumulative 44% is obtained by summing up the remaining peptide-loop interacting conformations).

**Figure 3:**
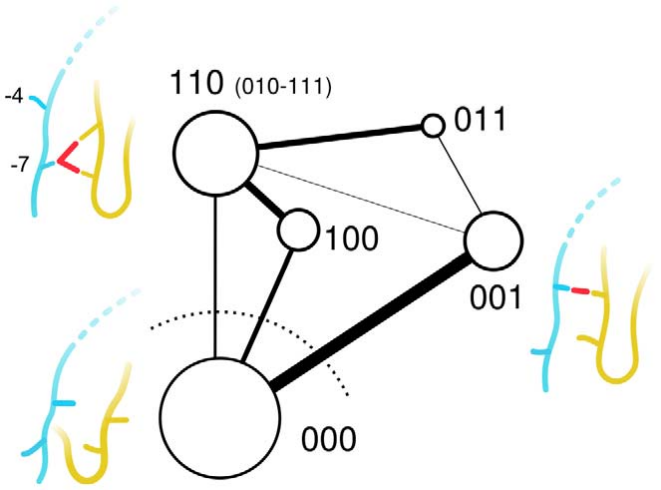
Transition network between the different peptide binding modes. The 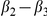 loop, the N-terminal part of the ligand and a schematic representation of the interactions between them are shown in yellow, light-blue and red, respectively. Node surface and edge thickness are proportional to the population of the configuration and the total transition probability, respectively. For each node, the three-letter string of the most populated configuration is indicated. Other minor configurations are shown in parenthesis, when present.

This scenario is represented in Fig. 3 by the transition network of the different peptide-loop configurations (see Methods). Multiple pathways are present, where a quite unspecific network of conformational changes stabilizes peptide-loop interactions on a time scale which is faster than unbinding (for example, 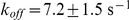 was measured for another member of the PDZ family [22]). Interestingly, the presence of peptide-loop interactions strongly influence the propensity to form canonical class-I contacts (see Fig. S2).

The dynamic nature of the interactions explains why peptide-loop contacts were difficult to detect by previous structural experimental investigations [1], [13]. For instance, both the original PDZ3 X-ray structure reported by McKinnon and collaborators [1] as well as further attempts by other groups (e.g. PDB-ID:1TP3) indicated that only a four residue C-terminal stretch (positions 0 to −3) is directly involved in binding. However, this observation is not supported by *in vitro* evolution and mutagenesis studies [7], [9], [15]. Along the same line, titration calorimetry experiments provided evidence for the role of peptide positions beyond −3 for both affinity and specificity [23], while water-mediated interactions were found when bound to the oncogenic E6 peptide [13]. Our observations reconcile these two views, providing a unifying picture for peptide binding to PSD95-PDZ3. While a dominant configuration characterizing the interactions between the peptide and the loop is absent, the cumulative effect of these interactions is necessary for binding. This effect is mostly dynamical, indicating that structure alone does not suffice to understand function in this case.

### Microscopic origin for the binding entropic penalty in the truncated form of PDZ3

**Figure 4:**
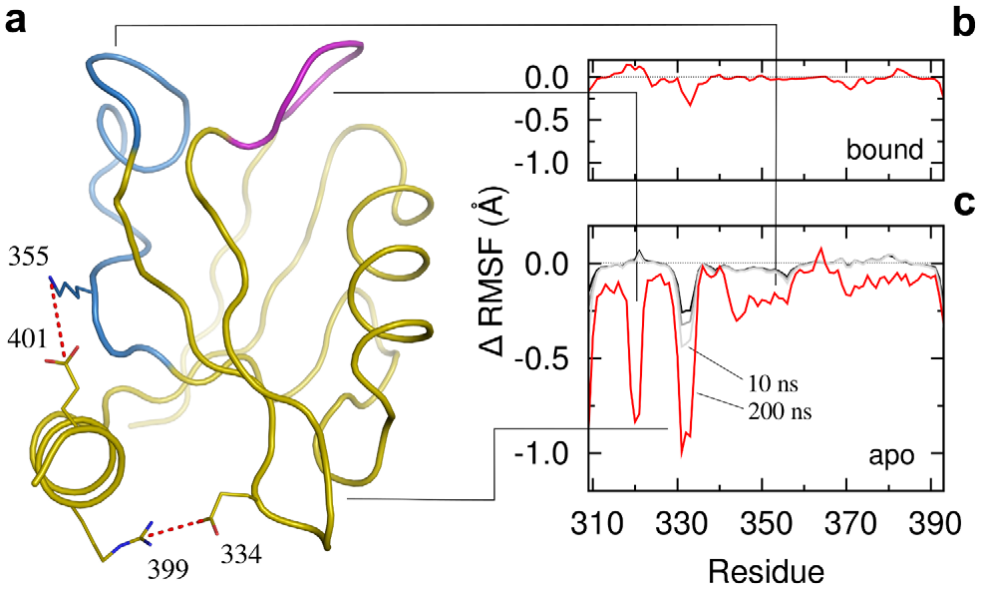
Extra-domain helix ionic interactions. (a) Two strong ionic interactions are formed between the helix and the PDZ domain (red dashed lines). Magenta and blue structures correspond to residues 318–323 (the carboxylate binding loop) and 342–357, respectively. (b) Backbone RMSF differences between the WT and the helix truncated form 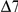 in the bound state. The RMSF time window is of 200 ns. (c) Backbone RMSF differences in the apo state. Red, light grey, grey and black lines correspond to RMSF calculated on time windows of 200, 10, 6 and 3 ns, respectively. The lack of the helix enhances the flexibility of both the 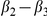 loop and the carboxylate binding loop. The latter (structure in magenta) couples with the region containing the helix interacting residue 355 (structure in blue). The enhanced backbone fluctuations do not appear at time scales faster than 10 ns (grey and black lines), the typical time range accessible by NMR spin relaxation techniques.

PSD95-PDZ3 is characterized by an extra-domain helix at the C-terminus [1], [17]. Structural analysis of our MD data showed that the helix directly interacts with the 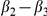 loop as well as with a region distant from the binding site, via two salt-bridges (red dashed lines in Fig. 4a). The first one involves 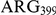 at the end of the helix and a negatively charged amino acid on the 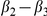 loop, 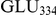. The second ionic interaction is between helix 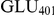 and 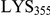, which is located in a region of the domain without specific secondary structure. This region (blue in Fig. 4a), in turn, is in spatial contact with the carboxylate binding loop. No specific helix-peptide interactions were found, only unstable hydrophobic contacts.

Recent experiments indicated that the extra-domain helix strongly influences the dynamics of the domain [17]. Binding affinity to the 9-mer CRIPT peptide was shown to decrease by 21-folds upon helix truncation through a purely entropic effect. The truncated form of PDS95-PDZ3 is defined by residues 306–395, and referred to as 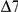 throughout the text [17]. To provide atomistic insights into this mechanism, MD simulations of 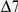 bound to CRIPT5 and CRIPT9 were performed (see Table. S1). The short 5-mer peptide unbound very quickly (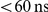) from the domain in all the four simulation runs, while CRIPT9 remained in the binding site. As observed for the WT, binding is stabilized by a network of dynamic salt-bridges between the ligand and the 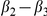 loop (see Fig. S2).

Analysis of the backbone root-mean-square-fluctuations (RMSF) in the WT showed that the flexibility of the bound form is not affected by helix truncation (Fig. 4b). However, it affects the unliganded (apo) form, enhancing the overall domain backbone flexibility (Fig. 4c). The enhanced flexibility is mainly localized in three regions: the carboxylate binding loop (residues 318–323), the 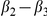 loop (residues 330–336) and residues 341–356. The latter corresponds to the region where the helix is forming the salt-bridges with 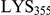. In our simulations for the WT, this interaction is present 49% and 41% of the time in the apo and bound forms, respectively. Given the spatial vicinity between this region (i.e., 341–356) and the carboxylate binding loop, we assume that the peaks relative to these two regions are coupled, arising from the missing interaction with the helix. Similarly, the enhanced flexibility of the 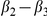 loop is induced by the missing interaction with the extra-domain helix through the salt-bridge between 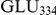 and 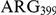. This interaction is very stable in both the apo and peptide-bound states, being formed 83% and 82% of the time, respectively.

These observations have important consequences for the interpretation of the entropic penalty upon binding to 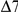. Given that the flexibility of the bound form is unaffected by helix truncation, while it is much larger in the apo form, peptide binding to 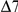 requires the quenching of the three regions reported in Fig. 4c and described above. Hence, our results suggest that the quenching of both the carboxylate and 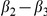 loops is responsible for the entropic penalty. Nevertheless, we cannot fully exclude other effects like a contribution from side chain dynamics, since decoupling entropy into local terms is a controversial and unsolved problem [24], [25].

The important role of backbone dynamics is in contrast with recent NMR relaxation experiments which found a negligible contribution of the backbone compared to side chains flexibility [17]. We suggest that this apparent contrast can be solved by looking at the time scales of the fluctuations reported in Fig. 4c. RMSF differences peaks vanish when the time windows used for the calculations are similar to the ones relevant for NMR 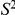 measurements, i.e. of the order of 10 ns or less (grey and black lines in Fig. 4c). Our data indicates that the relevant backbone fluctuations are on the 100 ns time scale. Such dynamics is, on the one hand too slow to be detected by NMR spin-relaxation techniques (i.e. 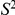) [19], [25], [26] and, on the other hand, too fast to show up as a separate subpopulation in NMR relaxation-dispersion experiments (i.e. 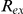).

### Hydrophobic stabilization of the extra-domain helical extension and the role of 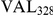


**Figure 5:**
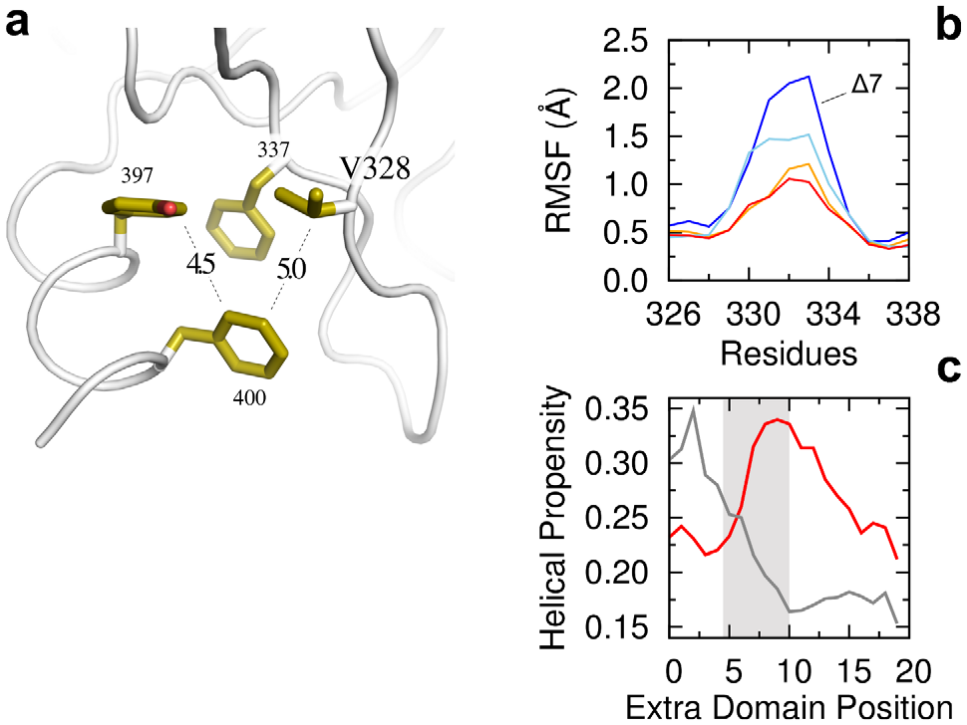
Extra-domain helix hydrophobic patch. (a) Four amino acids form the patch. Pairwise distances are on the order of 5 Å. (b) Backbone RMSF of the 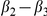 loop upon different mutations. WT is shown in orange. V328I and V328A mutants are displayed in red and light-blue, respectively. The helix truncated form 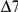 is shown in blue. (c) Helical propensities for the extra-domain C-terminal region of all human PDZ domains. Average helical propensity is displayed for domains with ILE/LEU/VAL at position 328 (red curve, average over 65 domains) and with ALA at position 328 (grey curve, 31 domains). The PSD95-PDZ3 extra-domain helix region is indicated as a grey box.

Stabilization of the extra-domain helix is further mediated by a hydrophobic patch, formed by 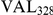 and 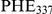 on the PDZ domain, and 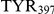 and 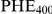 on the helix, as shown in Fig. 5a. Analysis of all human PDZ domains (see Methods) revealed that, while position 337 largely consists (i.e. 86%) of aliphatic or aromatic residues, position 328 is less conserved, with a large portion of aliphatic amino acids (see Fig. S3). Free-energy calculations between this helix and the PDZ domain performed with FoldX [27] (see Methods) predict that V328A and V328I mutants in the apo-form have a 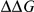 of 1.35 and −0.79 kcal/mol, respectively. Hence, mutation to ALA destabilizes the domain. MD simulations of both mutants are consistent with this scenario. Given the direct interaction between the extra-domain helix and the 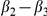 loop (Fig. 5b), it is found that bulkier aliphatics make this loop more rigid, avoiding the peptide induced quenching upon binding described in the previous section. Reversely, loop flexibility of the V328A mutant increases, approaching the one obtained in absence of the extra-domain helix (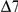, blue line). These results suggest a correlation between bulkier aliphatics at position 328 and the presence of an extra-domain helix.

To further investigate this hypothesis, we used PSIPRED [28] to compute the helical propensity of C-terminal segments in all 258 human PDZ domains (see Methods). A larger helical propensity is found for domains with ILE, LEU or VAL at position 328, compared to the ones with ALA (see Fig. 5c). For instance, around 10 residues downstream of the C-terminus, an helical propensity twice as large is found (P-value of 0.02, see Methods). These results correlate very well with our previous findings, indicating that large aliphatic side chains at position 328 can serve as anchors for extra-domain segments, stabilizing the 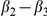 loop. Consequently, domains with an alanine at position 328 are less likely to have an extra-domain helix and we expect that in those cases the 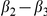 loop would be structured differently with respect to PSD95-PDZ3. This is in agreement, for example, with both PDZ1 and PDZ2 of PSD95. These domains are known to lack the C-terminal extra helix, possess an alanine at position 328 and have a different composition of the 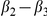 loop (see next section).

### Generalization to other PDZ domains

The PSD95-PDZ3 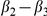 loop (together with V328) and the extra-domain alpha-helix are remarkably well conserved in orthologs up to fly (and even partially conserved in worm), as well as in human paralogs such as SAP97 (DLG1), PSD93 (DLG2) or SAP102 (DLG3), see Fig. S4. In particular, the three charged residues involved in peptide binding and helix contact are conserved in almost all cases, providing indirect evidence that the same loop-mediated protein/ligand recognition is taking place in distant organisms. This is not the case when looking at the entire PDZ family, where the 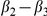 loop is highly heterogeneous both in length and amino acid composition. For instance, the loop of the PSD95-PDZ2 is more rigid, making self-interactions with the main domain body in a region close to the hydrophobic patch mentioned earlier [29]. Despite these differences, there are studies suggesting a role of the loop in binding to PDZ2. Large chemical-shifts were measured in the loop region upon binding, substantially contributing to affinity [29]. Finally, X-ray crystallography of PDZ2 from the human paralog DLG1 in complex with the oncogenic E6 peptide pointed out to an asparagine on the loop (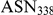) interacting with the ligand backbone at position 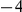 (using our notation) [13].

**Figure 6:**
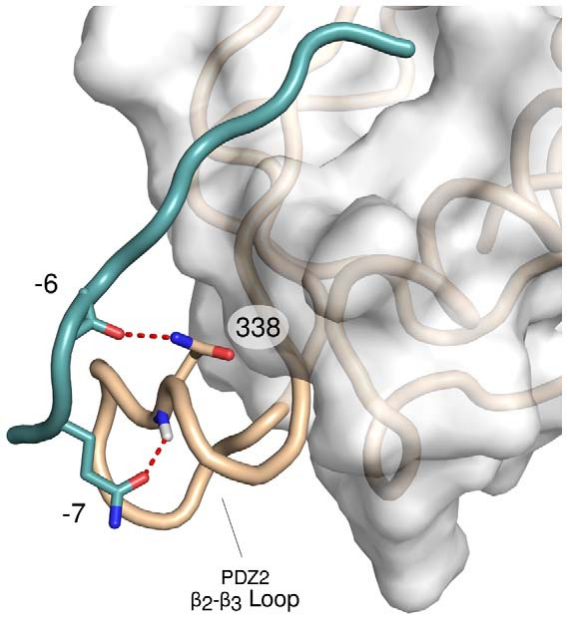
One of the predicted binding modes of the DLG1-PDZ2 domain in complex with the E6 peptide based on our MD simulations. The peptide interacts with the 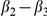 binding loop via two hydrogen bonds, involving the backbone oxygen of 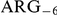 with the side chain of 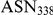 and the side chain of 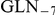 with the backbone nitrogen of 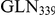. These two interactions occur simultaneously for 22% of the simulation time.

To provide a dynamical picture of the process, we performed additional simulations of the DLG1-PDZ2:E6 complex (see Methods). Our calculations reiterate the importance of 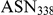 for binding to PDZ2. It is found that the E6 peptide is in contact with the loop through mainly three interactions, 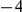: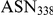, 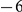: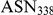 and 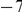: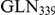, for a total of 69% of time. An example structure is shown in Fig. 6. These contacts interconvert on a ns time scale. Together with the results obtained for PDZ3, these observations suggest that the 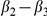 loop is actively involved in binding specificity: a property that would need to be consistently explored throughout the entire PDZ family.

## Discussion

In PDZ binding, the relatively limited information about peptide amino acids more distant from the C-terminus prevented a clear structural understanding of the effect and importance of these upstream side chains. Our work aims to fill this gap by providing calculations with both a canonical 5-mer CRIPT peptide as well as a longer 9-mer peptide in complex with PSD95-PDZ3. Three main results emerge from our work.

First, we observe in our simulations that peptide binding is mediated by ionic interactions with the loop following the binding site, referred to here as the 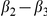 loop. These contacts are found with the 9-mer peptide, while the shorter 5-mer unbinds spontaneously after a few tens of ns. Recent experimental results on several PDZ domains support our interpretation [23], [30]. Strong differences between short and long peptides were found for negatively charged 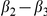 loops (e.g. MAGI1-PDZ2) [30]. Peptide-loop contacts are dynamic, where multiple specific interactions interconvert on a fast time scale of tens of ns (i.e. much faster than unbinding [22], [31]). Such dynamic interactions are likely to characterize several other PDZ domains. Further calculations on another member of the PDZ family, the DLG1-PDZ2, which is characterized by a different 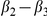 loop, support our hypothesis. Moreover, unresolved side chains away from the C-terminus are often found in other PDZ-ligand X-ray structures (see examples in Table S2), indicating that these side chains can adopt multiple conformations. We note that the presence of positively charged residues downstream of the fourth C-terminal positions of PDZ peptide ligands is well attested by recent experimental specificity profiles [7]. These charged residues are not necessarily always at the same positions, even within ligands of the same domain [30]. This is likely so because the peptide is flexible at these positions (as shown in Fig. 2). Consistently, 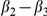 loops display a clear over-representation of negatively charged residues compared to other regions in PDZ domains: 11.6% of D/E in entire PDZ domains, 15.2% for D/E in 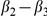 loops (according to the Fisher's test the probability to have this difference by chance is as low as 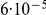, see Methods). Many of these residues on the loop provide clusters of negatively charged side chains that are ideally suited to recruit ligands with positive charges at any position between −4 and −7.

Second, we propose a mechanistic explanation for the microscopic origin of the binding entropic penalty in absence of the extra-domain helix of PSD95-PDZ3. In the apo form, the helix plays a crucial role in stabilizing both the carboxylate binding loop and the 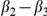 loop. Hence, these two loops are more flexible in the helix truncated domain. In this case, the peptide quenches the two regions upon binding, resulting in the observed entropic penalty. This quench does not take place when the extra-domain helix is present. Our findings suggest that extra-domain regions might play a more important role than mere linkers between functional domains [16], reiterating that the reductionist approach that protein domains can be studied in isolation should be always validated. This is especially important because several segments adjacent to domains show little sequence specificity (and thus are often not included in domain definition), although they adopt well-defined secondary structures such as the 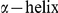 in the third PDZ domain of PSD95.

Third, analysis of 258 human PDZ domains as well as MD simulations of single-mutants allowed for the identification of an amino acid at the beginning of the 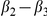 loop, VAL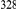 in PSD95, that correlates with the presence of the extra-domain helix in other PDZ domains. Prediction of helical propensities at positions following the C-terminus of the domain showed enhanced probability for those domains presenting bulkier aliphatic side chains other than alanine at that position. This analysis suggests that a binding mechanism, indirectly involving the extra domain helix as in PSD95-PDZ3, might be relevant for a significant portion of the PDZ domain family.

## Methods

### Simulations

Molecular dynamics simulations were performed using the GROMACS implementation [32] of the CHARMM27 force field [33], [34] at constant temperature and pressure with reference values equal to 300 K and 1 atm, respectively. The use of hydrogen virtual sites and fixed covalent bonds allowed a 4 fs integration time-step [35]. All systems were solvated in a dodecahedric box with an average of roughly 5000 tip3p water molecules (see Table S1 for details of each simulation setup). In the case of PDZ3, the system was equilibrated from the deposited X-ray structures 1BE9 and 1BFE [1] for the bound and apo forms, respectively, using residues 306–402 for the WT and 306–395 for 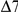. The PDZ2 starting structure is 2I0L [13] (from DLG1/SAP97). Each molecular setup was sampled by four independent runs of approximately 200 ns each for a total of 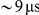 (Table S1). The first 50 ns of each trajectory were neglected in the analysis to reduce the bias from the starting configuration. Snapshots were saved every ps. The peptide N-terminus was neutralized in all cases, except CRIPT5*. The sequences of the 9-mer peptides are 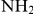-TKNYKQTSV-COOH and 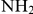-LQRRRETQV-COOH for PDZ3 and PDZ2, respectively. The first 5 peptide residues (i.e., positions from −4 to −8) as well as mutations at position 328 and the truncation of the extra domain helix were modeled using PyMol [36]. For each run, backbone RMSF values were calculated per residue as an average over the atoms C,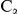 and N. Final RMSF values were averaged over the four runs. Molecular trajectories were analyzed with the programs WORDOM [37], [38] and GROMACS [39]. Hydrogen bonds were determined based on cutoffs for the angle Acceptor - Donor - Hydrogen (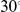) and the distance Donor - Acceptor (3.6 Å). Ionic interactions are considered to occur when the two last carbons before the charged atoms are closer than 5 Å.

### Structural analysis

Each protein-ligand snapshot was labeled by a *four*-digits code. The first three digits describe the peptide-loop interactions, e.g. “110”. The last digit represents an id, encoding the peptide structural conformation (i.e., the internal degrees of freedom). The latter was obtained by running a leader-based cluster-analysis on the ligand backbone (atoms C,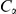 and N) with a 2 Å cutoff, using the program WORDOM [37], [38]. This digit distinguishes between different peptide conformations characterized by the same contacts with the loop. Each four-digit string represents a *microstate* of the protein-ligand complex. This decomposition is used to build a conformation-space-network [40]–[42], where each microstate is a node and a link between two nodes is placed if there is a direct transition between them during the MD simulation. Basins of attraction are defined using a gradient-cluster analysis [43], [44], where multiple microstates are lumped together if they interconvert rapidly. Each gradient-cluster represents a metastable configuration, which can contain heterogeneous peptide-loop contacts. Connectivity between these metastable configurations is represented as a coarse-grained network as shown in Fig. 3 (see also Fig. S2 in the Supp. Mat.). The gradient-cluster algorithm is freely available in the program PYNORAMIX (GPL license, available at the website raolab.com).

Predictions of free-energy differences upon mutations were done with FoldX using the BuildModel option after properly repairing the structures with the RepairPDB command [27]. The initial structure (PDB 1BFE) was first minimized with GROMACS in explicit water. This structure was originally crystallized with an ILE at position 328. We mutated it both to VAL (WT) and ALA to compute the free-energy differences.

### Human PDZ domain sequence analysis

The set of all human PDZ domains was retrieved from PFAM [45] and SMART [46] databases. A first multiple sequence alignment was generated with MUSCLE [47]. The alignment was manually curated, removing PDZ domains that could not be unambiguously aligned (most of them are unconventional PDZ domains). This resulted in a total number of 258 PDZ domains (see Table S3). The 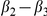 loop was mapped by homology starting form the structure of PSD95-PDZ3. Several PDZ domains are close paralogs, and this can result in strong biases when computing frequencies or correlation patterns. To account for this effect, we always grouped paralogs together (see Table S4). Groups of paralogs were defined using a cut-off of 50% on the sequence identity. The contribution of each member of a group was weighted by the inverse of the group size. For instance, to compute the amino acid frequency at a given position, residues from a group of 5 paralogs only contributed 1/5 each to the total frequencies. The helical propensity of C-terminal extensions of PDZ domains was computed with PSIPRED [28] for up to 20 residues downstream of the domains. If the protein C-terminus was reached before the 20 residues, a helix propensity of 0 was used. Here again, the contribution of paralogs was weighted to prevent purely phylogenetic correlations. P-values were computed by reshuffling the amino acid composition at position 328 in all PDZ domains of Table S3. The Fisher's test was used to compute the probability to have a given number of negative residues within all loop residues, knowing the total number of negative residues within the sequences of all PDZ domains [48].

## Supporting Information

Figure S1MD simulation time series of the PDZ3 complexed with the 5-mer CRIPT peptide. A rebinding event of the 5-mer CRIPT peptide to PDZ3 is highlighted in grey. (Top) Distance between the 5-mer peptide side chain nitrogen of 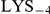 and the 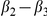 loop 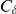 of 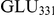. (Bottom) Backbone RMSD from the X-ray structure of peptide residues 0:−4 for the 5-mer and the 9-mer peptides are shown in blue and black, respectively. The longer peptide stays tightly bound for the whole simulation time (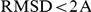). The short peptide immediately goes into a partially unbound state (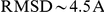), unbinding completely after roughly 110 ns. At 50 ns a rebinding event occurs (grey areas). During this event the two charged side chains of 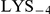 and 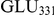 come closer, suggesting that the rebinding process is mediated by this ionic interaction.(TIF)

Figure S2CRIPT peptide affinity scheme for PSD95-PDZ3. (Top) Schematic representation of the domain-peptide complex. Experimental binding affinities from Ref. [17] are reported (no affinities are found for 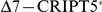, indicated with a *). (Middle) Transition network between the different peptide binding modes (see main text for details). Pie charts surfaces indicate the population ratio between the different configurations. Interactions involving exclusively 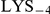 or 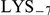 are indicated in pink and red, respectively. In dark red both lysines are engaged with the loop, white for no interactions. (Bottom) The distribution of the total number of canonical interactions for each complex. We monitored three key contacts. They are the hydrogen bond between 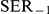 and the side chain oxygen of 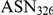; the hydrogen bond between the hydroxyl oxygen of 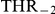 and 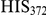 (a milestone for PDZ specificity); the hydrogen bond between 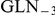 and the side chain oxygen of 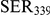.(TIF)

Figure S3 Amino acid frequencies at position 328 in human PDZ domains. Contribution of paralog domains have been weighted as described in Methods.
(TIF)

Figure S4Conservation of the 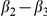 loop and extra-domain alpha helix in PSD95-PDZ3 orthologs. Green shading corresponds to biochemically similar side chains. Orange shading corresponds to non-conserved residues.(TIF)

Table S1 Details of the simulations performed.
(PDF)

Table S2 Examples of PDZ structures in complex with ligands with unresolved residues between position −4 and −7.
(XLSX)

Table S3 Manually curated multiple sequence alignment of the 258 human PDZ domains used in this work.
(XLSX)

Table S4 Clusters of human PDZ paralogs.
(XLSX)
