# Supplementary figures and images for: Beyond the Binding Site: The Role of the β2 – β3 Loop and Extra-Domain Structures in PDZ Domains
Source: PLoS Comput Biol. 2012 Mar 8;8(3):e1002429. doi: 10.1371/journal.pcbi.1002429 (PMC3297566; doi:10.1371/journal.pcbi.1002429)

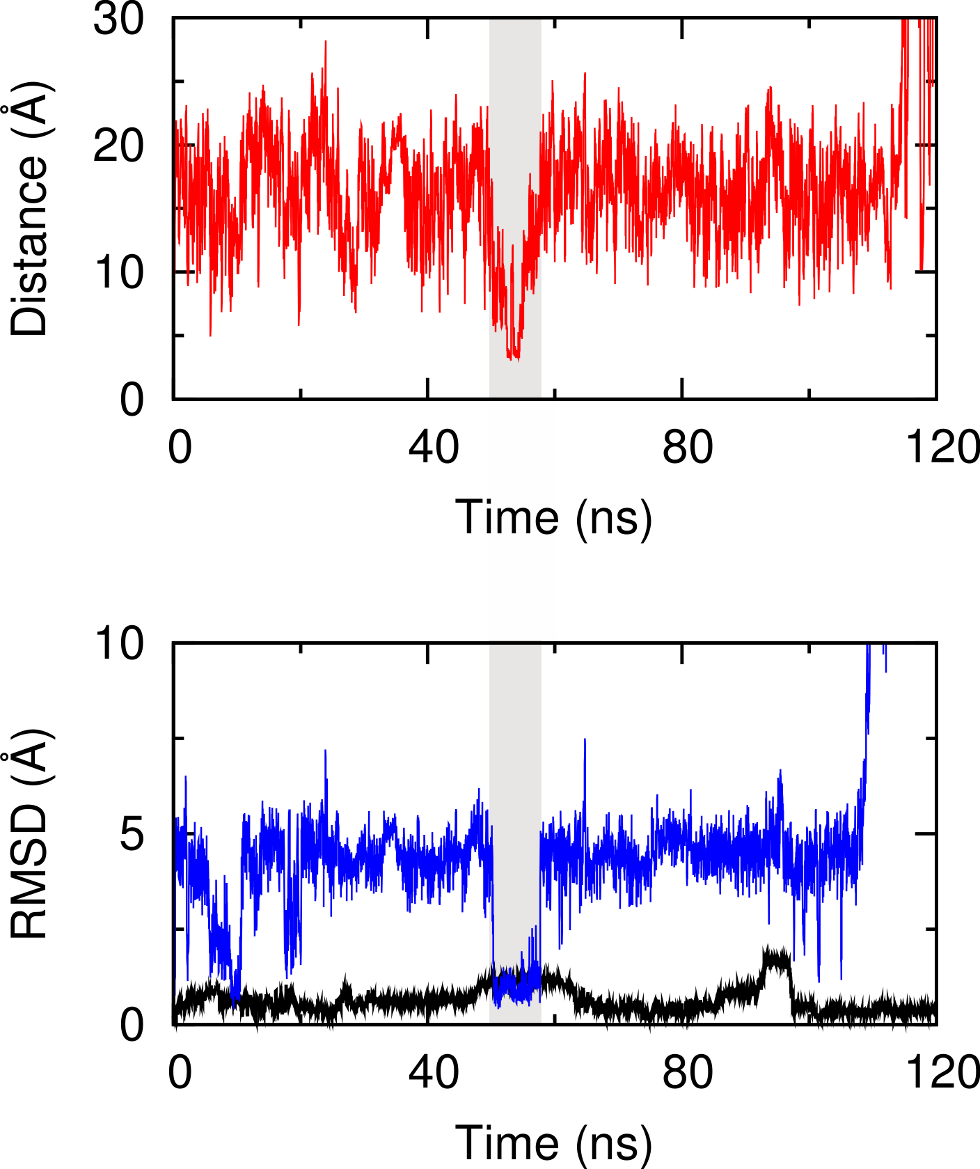

Supplement: Figure S1 — MD simulation time series of the PDZ3 complexed with the 5-mer CRIPT peptide. A rebinding event of the 5-mer CRIPT peptide to PDZ3 is highlighted in grey. (Top) Distance between the 5-mer peptide side chain nitrogen of and the loop of . (Bottom) Backbone RMSD from the X-ray structure of peptide residues 0:−4 for the 5-mer and the 9-mer peptides are shown in blue and black, respectively. The longer peptide stays tightly bound for the whole simulation time (). The short peptide immediately goes into a partially unbound state (), unbinding completely after roughly 110 ns. At 50 ns a rebinding event occurs (grey areas). During this event the two charged side chains of and come closer, suggesting that the rebinding process is mediated by this ionic interaction. (TIF) [file pcbi.1002429.s001.tif]

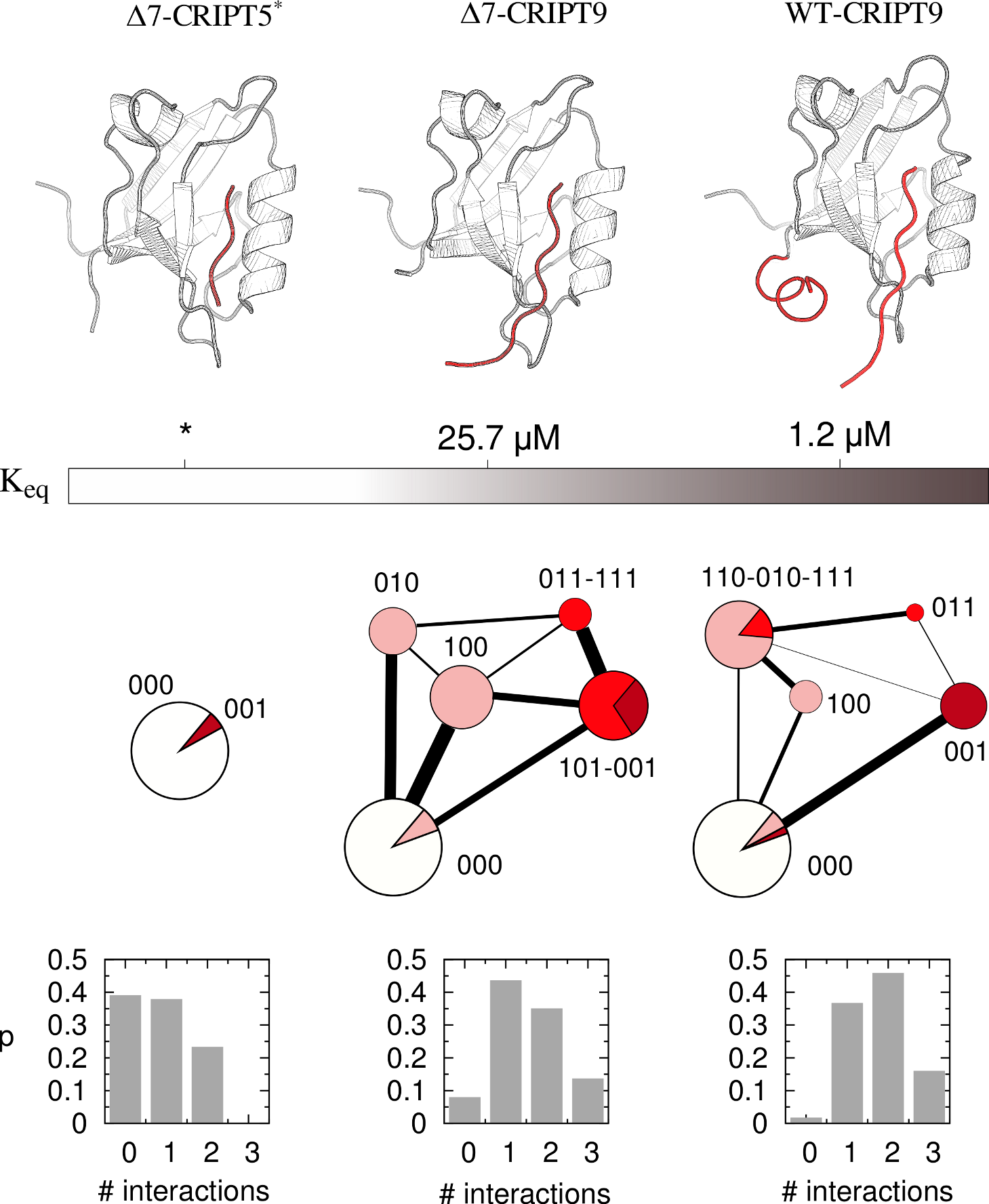

Supplement: Figure S2 — CRIPT peptide affinity scheme for PSD95-PDZ3. (Top) Schematic representation of the domain-peptide complex. Experimental binding affinities from Ref. [17] are reported (no affinities are found for , indicated with a *). (Middle) Transition network between the different peptide binding modes (see main text for details). Pie charts surfaces indicate the population ratio between the different configurations. Interactions involving exclusively or are indicated in pink and red, respectively. In dark red both lysines are engaged with the loop, white for no interactions. (Bottom) The distribution of the total number of canonical interactions for each complex. We monitored three key contacts. They are the hydrogen bond between and the side chain oxygen of ; the hydrogen bond between the hydroxyl oxygen of and (a milestone for PDZ specificity); the hydrogen bond between and the side chain oxygen of . (TIF) [file pcbi.1002429.s002.tif]

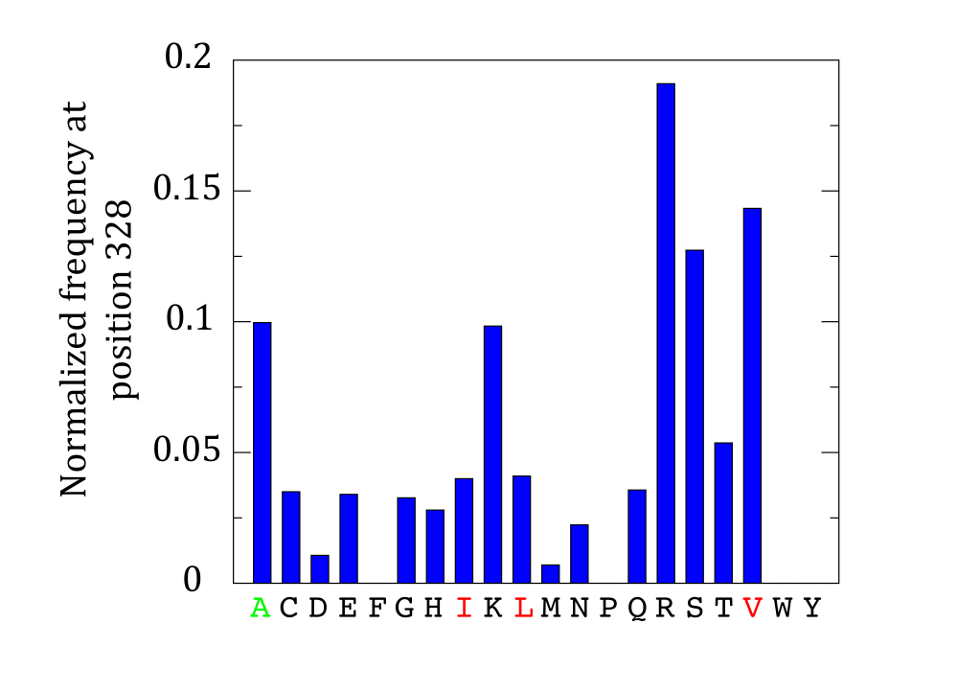

Supplement: Figure S3 — Amino acid frequencies at position 328 in human PDZ domains. Contribution of paralog domains have been weighted as described in Methods. (TIF) [file pcbi.1002429.s003.tif]

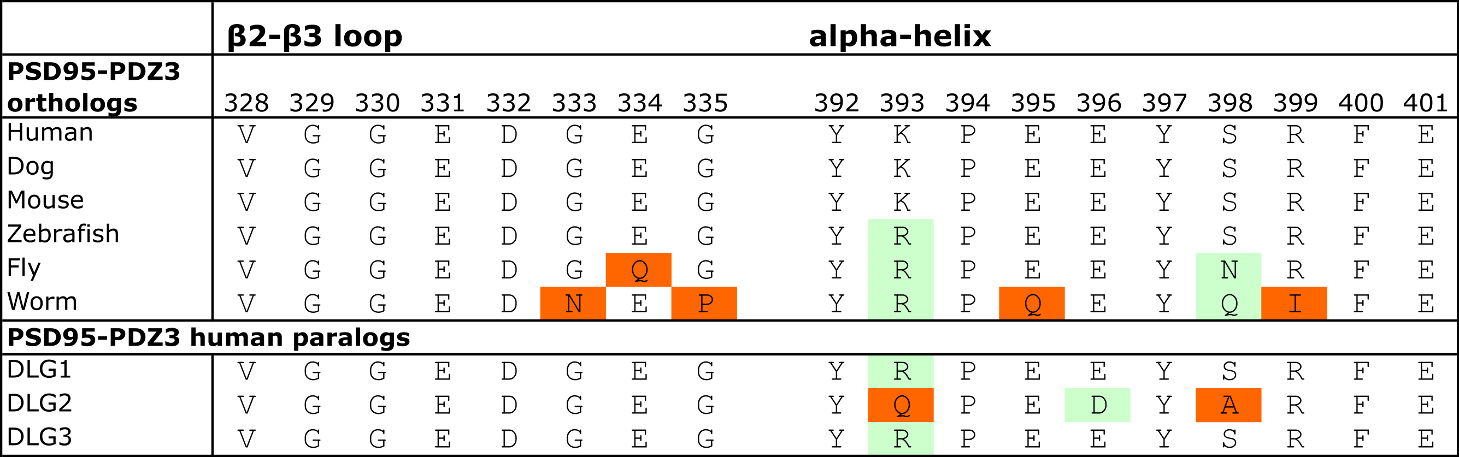

Supplement: Figure S4 — Conservation of the loop and extra-domain alpha helix in PSD95-PDZ3 orthologs. Green shading corresponds to biochemically similar side chains. Orange shading corresponds to non-conserved residues. (TIF) [file pcbi.1002429.s004.tif]
