# Supplementary material for: Beyond the Binding Site: The Role of the β2 – β3 Loop and Extra-Domain Structures in PDZ Domains
Source: PLoS Comput Biol. 2012 Mar 8;8(3):e1002429. doi: 10.1371/journal.pcbi.1002429 (PMC3297566; doi:10.1371/journal.pcbi.1002429)
Supplement: Table S1 — Details of the simulations performed. (PDF) [file pcbi.1002429.s005.pdf]

name	# runs	Tot. Sim. Time (μs)	Unbinding Times (ns)	# H_2O
$PDZ3$ CRIPT5	4	0.424	13, 34, 65, 108	4699
$PDZ3$ CRIPT9	4	0.697	-	6001
$PDZ3_{apo}$	4	0.918	-	4385
Δ7 CRIPT5	4	0.262	19, 22, 45, 57	4596
Δ7 CRIPT5*	4	0.792	-	4597
Δ7 CRIPT9	4	0.705	-	6027
$\Delta 7_{apo}$	4	0.934	-	4425
$V328I$	4	0.787	-	5999
$V328I_{apo}$	4	0.917	-	4388
$V328A$	4	0.773	-	6000
$V328A_{apo}$	4	0.987	-	4389
PDZ2	3	0.579	-	6750
Total	47	8.775		
